# Do Coffee Polyphenols Have a Preventive Action on Metabolic Syndrome Associated Endothelial Dysfunctions? An Assessment of the Current Evidence

**DOI:** 10.3390/antiox7020026

**Published:** 2018-02-04

**Authors:** Kazuo Yamagata

**Affiliations:** Laboratory of Molecular Health Science of Food, Department of Food Bioscience and Biotechnology, Nihon University College of Bioresource Sciences (NUBS), 1866, Kameino, Fujisawa, Kanagawa 252-8510, Japan; kyamagat@brs.nihon-u.ac.jp; Tel.: +81-466-84-3986

**Keywords:** antioxidative effects, chlorogenic acid, endothelial dysfunction, metabolic syndrome

## Abstract

Epidemiologic studies from several countries have found that mortality rates associated with the metabolic syndrome are inversely associated with coffee consumption. Metabolic syndrome can lead to arteriosclerosis by endothelial dysfunction, and increases the risk for myocardial and cerebral infarction. Accordingly, it is important to understand the possible protective effects of coffee against components of the metabolic syndrome, including vascular endothelial function impairment, obesity and diabetes. Coffee contains many components, including caffeine, chlorogenic acid, diterpenes and trigonelline. Studies have found that coffee polyphenols, such as chlorogenic acids, have many health-promoting properties, such as antioxidant, anti-inflammatory, anti-cancer, anti-diabetes, and antihypertensive properties. Chlorogenic acids may exert protective effects against metabolic syndrome risk through their antioxidant properties, in particular toward vascular endothelial cells, in which nitric oxide production may be enhanced, by promoting endothelial nitric oxide synthase expression. These effects indicate that coffee components may support the maintenance of normal endothelial function and play an important role in the prevention of metabolic syndrome. However, results related to coffee consumption and the metabolic syndrome are heterogeneous among studies, and the mechanisms of its functions and corresponding molecular targets remain largely elusive. This review describes the results of studies exploring the putative effects of coffee components, especially in protecting vascular endothelial function and preventing metabolic syndrome.

## 1. Introduction

Metabolic syndrome is a combination of medical conditions, including dyslipidemia, elevated blood pressure, insulin resistance, and excess body weight. An increase in the rate of adult obesity has led to increases in obesity-associated metabolic disorders, such as insulin resistance, glucose intolerance, dyslipidemia, and hypertension, which are major risk factors for cardiovascular disease (CVD) [1]. While the mechanism underlying metabolic syndrome is poorly understood, insulin resistance appears to be an important factor [2]. The occurrence of hyperglycemia, dyslipidemia, and hypertension in metabolic syndrome is associated with endothelial dysfunction and promotion of atherogenesis [3]. These changes show that patients with metabolic syndrome have an increased risk of myocardial infarction and stroke [4,5], which, together, contribute to the burden of non-communicable diseases [6]. 

Various components of the common diet have been suggested to potentially affect endothelial function, including fruits, vegetables, olive oil and nuts [7,8,9,10]. Several epidemiological studies have shown that coffee consumption is associated with a lower risk of cardiovascular disease, metabolic disorders and certain cancers [11]. Among the observed results, evidence suggests that individuals consuming moderate amounts of coffee might be less likely to be affected by metabolic syndrome [12,13]. Evidence has been increasing recently supporting the protective effects of coffee and its components, such as caffeine, chlorogenic acids and diterpenes, against oxidative stress and related metabolic syndrome risk [14,15]. Results from a recent meta-analysis indicated that the coffee intake was inversely related with the risk of type 2 diabetes (T2DM) [16]. These effects are likely due to the presence of chlorogenic acids and caffeine [17]. Moreover, other reports described the effect of coffee intake on lipid, protein and DNA damage, and the modulation of antioxidant capacity and antioxidant enzymes in human studies [18,19]. These results suggested that coffee consumption enhances glutathione levels and offers protection against DNA damage [19,20]. In contrast, caffeine consumption of ≥6 units/day during pregnancy is related with impaired fetal length growth [21]. Also, this report indicated that a higher caffeine intake might preferentially adversely affect fetal skeletal growth.

In metabolic syndrome, inflammation is promoted, which strongly increases the risk of systemic CVD [22,23]. Endothelial dysfunction reduces vasodilation, enhances proinflammatory status and thrombogenesis, and is the first step in the development of CVD [24]. The development of arteriosclerosis that occurs in metabolic syndrome is attributed to endothelial dysfunction. Namely, these events of endothelial dysfunction strongly induce CVD [25]. On the other hand, the intake of coffee contributes to the prevention of endothelial dysfunction and decrease in CVD. A recent study demonstrated that the inverse relationship between coffee consumption and metabolic syndrome may reflect coffee’s content of antioxidants that offer cardiovascular protection [26]. This review considers the effects of coffee polyphenols on vascular endothelial function in preventing metabolic syndrome. The association of coffee intake with metabolic syndrome and with vascular disorders induced in metabolic syndrome will be discussed.

## 2. Antioxidant Effects of Coffee Components

Coffee is a major source of antioxidant polyphenols in the Japanese diet [27]. As shown in Table 1, coffee has the highest total polyphenol content in beverages, followed by green tea.

Coffee contains over 1500 chemicals, most of which are formed during the roasting process. The total polyphenol consumption from coffee is 426 mg/day, which makes up 50% of the amount of polyphenols consumed daily in the Japanese population [27]. Typically found polyphenols include caffeine, chlorogenic acid, diterpenes and trigonelline [28]. The chemical composition of green coffee beans is shown in Table 2 [29]. The main water-soluble constituents in coffee are phenolic polymers, polysaccharides, chlorogenic acids, minerals, caffeine, organic acids, sugars, and lipids. Furthermore, the lipid-soluble constituents of coffee include mainly triacylglycerols, tocopherols, and esters of diterpene alcohols with fatty acids.

Coffee is a major source of antioxidant polyphenols in the Japanese diet [28]. Coffee has antioxidative components and contributes to oxidative stress prevention [30]. The antioxidative compounds inhibit nicotinamide adenine dinucleotide phosphate (NADPH) oxidase in the mitochondria and decrease production of reactive oxygen species (ROS) [31]. For this reason, coffee is understood to be a diet beverage that can inhibit oxidative stress. The coffee component induces antioxidant activity and endothelial nitric oxide (NO) production. For example, intake of caffeoylquinic (CQA, a representative of chlorogenic acid) for 8 weeks decreased NADPH-dependent ROS production and enhanced NO production in the aorta of spontaneously hypertensive rats [32]. In addition, CQA was directly related to the blocking of gene expression of the NADPH-oxidase component, p22phox [32]. These results indicate that the CQA might decrease oxidative stress and induce NO production and help to prevent endothelial dysfunction, such as vascular hypertrophy and hypertension, in spontaneously hypertensive rats.

**Figure 1:**
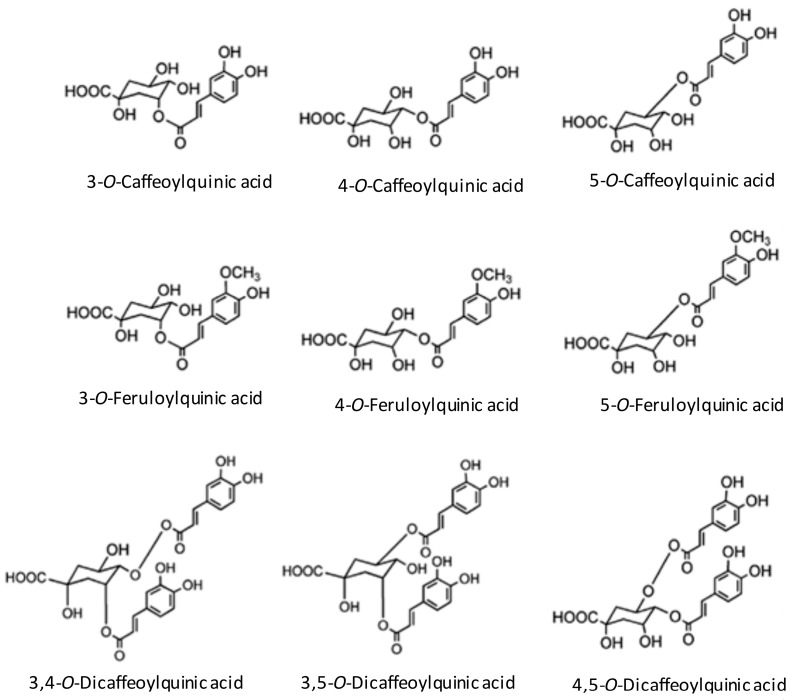
Structure of main chlorogenic acids in coffee (Adapted from reference Stamach et al., 2006) [29].

The main antioxidants in coffee are the chlorogenic acids, caffeine, and melanoidins [17]. Chlorogenic acids consist of a family of esters formed between quinic acid and caffeic acid. The subclasses of chlorogenic acids are CQA, feruloylquinic (FQA) and dicaffeoylquinic (diCQA) acid. The main chlorogenic acid subclass in coffee is CQA [17,33] (Figure 1). Following acute consumption of coffee, a significant 5.5% rise in plasma antioxidant activity in human has been demonstrated [34], including inhibition of low density lipoprotein (LDL) oxidation [35,36]. Therefore, the effect of CQA may contribute to an arteriosclerotic preventive mechanism. Also, caffeine has antioxidant properties, such as the ability to scavenge hydroxyl radicals [37]. Caffeine has been shown to scavenge superoxide radicals by measurements of O_2_^−^ after reaction with caffeine using electron paramagnetic resonance (EPR) [38,39]. These results support the findings of another study in which caffeine had antioxidative effects and inhibited peroxidation of LDL [40]. Finally, the browned material in coffee also has antioxidative effects. The taste and the color of the coffee are produced mainly by a roasting process with the Maillard reaction. These substances contribute to the antioxidation effect and oxidative stress of the coffee [41,42]. In the roasted coffee bean, melanoidin is produced from nonenzymatic browning and accounts for the coffee bean’s antioxidant activity [43]. Chlorogenic acid decreases in roasting, but melanoidins increase and may make up for the decrease in antioxidation from a loss of chlorogenic acid [44]. The antioxidative difference of the coffee changes by a roast. Differences in reactivity for roasts on antioxidants, such as chlorogenic acid, may influence the antioxidation characteristics of the coffee. 

## 3. Epidemiological Studies of Coffee Consumption and the Metabolic Syndrome

### 3.1. Coffee Intake and Metabolic Syndrome

Regular consumption of coffee has been associated with lower odds of having metabolic syndrome [12,13]. Clinical evidence of the effects of coffee consumption on various components of the metabolic syndrome has been provided by a cross-over, randomized controlled study, investigating men and women with normal cholesterol levels (*n* = 25) and those with hypercholesterolemia (*n* = 27) aged 18–45 years with body mass index (BMI) ranging from 18–25 kg/m^2^. For 8 weeks, the study subjects consumed three servings/day of a blend providing coffee polyphenols, hydroxycinnamic acids (510.6 mg) and caffeine (121.2 mg), or a control drink. In the coffee consumption groups, blood pressure, body fat percentage, and levels of leptin, plasminogen activator inhibitor-1 (PAI-1) and resistin were reduced. In addition, glucose concentration, insulin resistance and triglyceride levels were reduced. Notably, these reductions were much greater in the group with hypercholesterolemia compared with the controls. These results suggest that regular coffee consumption can improve the pathologic condition of patients with metabolic syndrome-associated hypercholesterolemia.

Most of the existing evidence relies on the results of two meta-analyses showing an association between coffee consumption and metabolic syndrome in observational studies [12,13]. The meta-analyses included 13 studies with a total of 159,805 participants and showed an inverse association between regular coffee consumption and metabolic syndrome, despite evidence of heterogeneity between results of the studies included. The causes for these differences may have been variations across the studies in terms of lifestyle and the percentages of patients with metabolic syndrome as well as in coffee-drinking habits, such as adding milk, full-fat cream or sugar [45]. Several other factors may have accounted for the heterogeneity among results. For instance, most of the studies did not consider methods of preparation, type, and roasting process of coffee, which have been shown to influence the phytochemical component of the beverage [46]. Moreover, collinearity may exist with the intake of certain foods, sugar, and the presence or absence of milk [47]. Other sources of heterogeneity may be derived from lifestyle differences among individuals, for instance, the level of smoking, which has been shown to be an effect modifier of the association between coffee intake and health outcomes [48]. Third, none of the studies took into account genetic factors, which have been reported to affect the relationship between coffee and cardiovascular outcomes due to polymorphisms related to caffeine metabolization [49,50].

Collectively, it was shown that the association between coffee consumption and occurrence of metabolic syndrome varied greatly across studies (Table 3). In a large general population cohort study, high coffee consumption was associated with low risks of obesity, metabolic syndrome and T2DM [51]. The study results indicated that high coffee consumption was associated with decrease in obesity, metabolic syndrome and T2DM. Moreover, high coffee consumption was associated with low BMI, weight, height, systolic/diastolic blood pressure, triglycerides and cholesterol. In another study, the effects of coffee consumption in metabolic syndrome were investigated in healthy subjects: 174 men and 194 women were followed from the age of 27 years onwards [52]. This study began in 1977, along with an observational longitudinal study that examined 600 girls and boys. The strongest evidence supporting a positive health effect of coffee consumption has been for diabetes. However, this study demonstrated that long-term coffee consumption was not associated with metabolic syndrome. While coffee consumption appeared to be significantly reversely correlated with blood pressure, the relationship was no longer significant after adjustment for lifestyle covariates. In a Mendelian randomization study, we examined the relationship between coffee intake and obesity, metabolic syndrome, and T2DM in 93,179 people with T2DM in two large cohorts. A high intake of coffee was associated with a reduced risk of obesity, metabolic syndrome, and type II diabetes mellitus. Furthermore, higher coffee consumption was associated with reduced BMI, weight, waist circumference, blood pressure, triglycerides and total cholesterol and increased high-density lipoprotein cholesterol [51]. In addition, a study of metabolic syndrome in Poland investigated the association of tea and coffee consumption with the prevalence of metabolic syndrome in 8821 subjects aged 20 years and older. The relationship of coffee intake with metabolic syndrome suggested a role for coffee intake in cardiovascular prevention. The effect of the coffee may have been caused by antioxidant action [53]. Still another study showed that the risk of metabolic syndrome was associated with coffee intake in 15,691 Korean women, indicating that coffee intake might be related to a decreased occurrence of metabolic syndrome in this population [54].

Furthermore, the report showed a relationship between coffee intake and metabolic syndrome in overweight and normal individuals. The studies included a questionnaire-style interview, blood pressure measurements and examination of fasting blood samples. In the obese and overweight groups, lower coffee intake compared with higher intake was associated with a higher risk of abdominal obesity, hypertension, abnormal glucose concentration, triglycerides and metabolic syndrome [55]. Another study examined the relationship between dietary lifestyle factors with metabolic syndrome. Daily drinking of 2–3 cups of coffee was inversely related to metabolic syndrome, and sleeping 7–8 h per night was related to decreased odds of metabolic syndrome [15]. In a cross-sectional study, coffee intake was inversely associated with metabolic syndrome and triglyceride levels in 1886 Italian subjects [55]. Also, in 8821 Italian subjects, coffee consumption was negatively associated with metabolic syndrome, waist circumference, hypertension and triglyceride levels [55]. The report also demonstrated that coffee intake was negatively associated with metabolic syndrome, waist circumference, hypertension and triglycerides [56]. Furthermore, in 17,953 Korean adults, when comparing those who consumed instant coffee >3 times/day with those who consumed instant coffee <1 time/week, the odds ratio for metabolic syndrome was 1.37. In addition, coffee drinkers had an increased risk of obesity, abdominal obesity and low levels of high-density lipoprotein [61]. In these studies, most of the subjects consumed milk or consumed an instant coffee mix containing sugar and powdered creamer. These results indicate that instant coffee drinkers have increased risks of these metabolic conditions, and that the consumption of the instant coffee mixture may have a noxious effect on metabolic syndrome. Therefore, the increased risk of metabolic syndrome may be attributed in part to the excessive intake of sugar and powdered creamer. Furthermore, the association between coffee and metabolic syndrome was evaluated by several large-scale prospective studies, despite results being mostly contrasting (9514 in the United States, [61]; 17,014 in Norway, [62]; and 368 in the Netherlands, [46]).

### 3.2. Coffee Intake and Obesity

The rate of obesity has increased on a global scale in both adults and children, is related to several comorbidities, such as hypertension and T2DM, and is strongly related to the onset of CVD [64]. In the United States, prospective studies examined the interaction of habitual coffee consumption with the genetic predisposition to obesity in relation to BMI in 5116 men and 9841 women [65]. Higher coffee consumption appeared to reduce the genetic association between obesity and BMI—individuals with greater genetic predisposition to obesity were observed to have a lower BMI, related to higher coffee consumption. Furthermore, a randomized clinical trial was performed with obese women aged 20–45 years in which an intervention group received 400 mg of coffee [66]. The body weight, body mass and fat mass indices, and waist-to-hip circumference ratio of the intervention group decreased, compared to the control group. Furthermore, the intervention group had decreased cholesterol and low-density lipoprotein (LDL) levels, compared to the control group. In contrast, the serum adiponectins increased in the intervention group. These results indicate that consumption of coffee may reduce obesity.

### 3.3. Coffee Intake and Type 2 Diabetes

A meta-analysis of prospective studies (10 articles involving 491,485 participants, including 29,165 with T2DM) was performed to evaluate the relationship between coffee and caffeine consumption and T2DM incidence [67]. The relative risk of T2DM decreased with coffee intake. In addition, a relationship between T2DM incidence and coffee intake was found among non-smokers with BMI <25 kg/m^2^. In particular, the effect on T2DM risk was higher in women. Furthermore, a large-scale case-cohort study demonstrated evidence for an interaction of incretin-associated TCF7L2 genetic variants and an incretin-specific genetic risk score with coffee consumption in relation to T2DM risk [68]. The cohort study included 11,035 participants, among them, 8086 incident T2DM cases. However, none of these relationships were statistically significant.

In cohort study of 2332 Chinese subjects, coffee intake was indicated to be inversely related to T2DM [69]. Habitual coffee consumption was associated with a 38–46% reduced risk of T2DM, compared to than non-drinkers. In a prospective cohort study in 88,259 US women (younger and middle-aged, incident cases 1263) were examined for coffee and caffeine intake and risk of T2DM [70]. The relative risk of T2DM decreased depending on coffee intake. Coffee consumption may decrease the risk of T2DM in younger and middle-aged women. Furthermore, reports indicated that the consumption in adults of up to 3 cups a day of coffee decreased the risk of T2DM and of metabolic syndrome [71]. Recently, in a Mendelian randomization study, habitual coffee consumption was inversely associated with T2DM, along with depression and Alzheimer’s disease onset [72]. These results indicate that coffee consumption may contribute to decreases in T2DM.

### 3.4. Coffee Intake and Non-Alcoholic Steatohepatitis

The relationship between non-alcoholic steatohepatitis with coffee intake was explored by several epidemiologic studies. One prospective cohort study indicated an inverse association between coffee consumption and liver cirrhosis [73,74]. This study involved a cohort of 63,275 Chinese subjects (middle-aged and older) in the Singapore Chinese Health Study [75]. Compared to non-daily coffee drinkers, those who drank two or more cups per day had a 66% reduction in mortality risk. Coffee intake was related to a decrease in the risk of mortality, except coffee intake was not associated with hepatitis B-related cirrhosis mortality. Furthermore, a cross-sectional study (*n* = 347) showed an inverse association between coffee consumption and liver fibrosis [76]. In the study, high coffee consumption was related to a lower occurrence of clinically significant fibrosis. This result suggests that coffee consumption may exert beneficial effects on fibrosis progression [77]. 

However, neither the occurrence of fatty liver, nor the prevalence of fatty liver, as assessed by ultrasonography (SteatoTest) and the hepatorenal index, were related to coffee consumption. In a cross-sectional study, the effects of coffee consumption were investigated in patients (*n* = 1018) with non-alcoholic fatty liver disease (*n* = 155), hepatitis C virus (*n* = 378), and hepatitis B virus (*n* = 485). Drinking two or more cups of coffee per day was related to improvements in pathologic conditions [77]. In an epidemiological study on the association of coffee intake with chronic liver disease, 286 patients in a liver outpatient department in a hospital in Scotland completed a questionnaire regarding coffee consumption and lifestyle factors. The results indicated that coffee intake may be related to a reduced prevalence of cirrhosis in patients with chronic liver disease [78].

### 3.5. Coffee Intake and Atherosclerosis

A cohort study in Tokushima Prefecture, Japan, investigated the relationship between coffee intake and arterial stiffness [79]. A report indicated that the intake of coffee was inversely related to arterial stiffness in 540 Japanese men [79]. Coffee intake was inversely related to arterial stiffness, independent of atherosclerotic risk factors. This result was related partially to a decrease in circulating triglycerides. In addition, other studies suggest that the addition of milk may affect coffee’s preventive action on arteriosclerosis [46,51]. On the other hand, in a cohort study, no association was observed between coffee or caffeine intake and coronary and carotid atherosclerosis. The Coronary Artery Risk Development in Young Adults (CARDIA) study examined the relationship between coffee intake and atherosclerosis in 5115 young adults [80]. No relationship was observed between atherosclerosis and the intake of average coffee, decaffeinated coffee, or caffeine intake. Furthermore, in 6508 ethnically diverse participants, coffee intake (>1 cup per day) was not associated with coronary artery calcification or cardiovascular events [81]. In a cross-sectional study, 1929 participants without known coronary heart disease, coffee intake and calcified atherosclerotic plaques in the coronary arteries were examined [82]. The results did not support a relationship between coffee intake and coronary-artery calcification in men and women. On the other hand, caffeine consumption was marginally inversely related to coronary artery calcification. As for the intake of more than 1 cup of coffee per day, caffeine may be related to cardiovascular events. In addition to epidemiological studies, further interventional studies may be needed to confirm the causal association.

### 3.6. Coffee Intake and Hypertension

In a meta-analysis of seven cohort studies, including 205,349 individuals and 44,120 cases of hypertension, an increase in 1 cup/day of coffee consumption was associated with a 1% decreased risk of hypertension [83]. Results from individual cohort studies suggest that the risk of hypertension depends on the coffee intake level. In a prospective cohort study of 24,710 Finnish subjects with no history of antihypertensive drug treatment, coronary heart disease, or stroke at baseline, the association between coffee intake and the incidence of antihypertensive drug treatment was investigated. The multivariate-adjusted hazard ratios for the amount of coffee consumed daily were marginally significant for baseline systolic blood pressure [84]. Low-to-moderate coffee intake appeared to increase the risk of antihypertensive drug treatment. In a cross-sectional population-based study including 8821 adults (51.4% female) in Poland, coffee consumption was negatively related to hypertension [85]. More details on the relationship of coffee intake with hypertension will have to be determined in future studies.

## 4. Coffee Composition and Features

**Figure 2:**
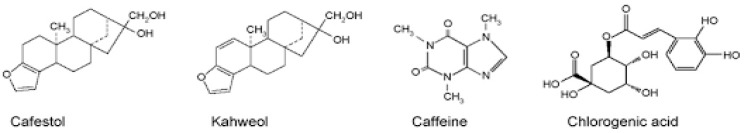
Chemical structures of proposed bioactive compounds in coffee (Adapted from reference Bonita et al., 2007) [86].

Commercially important coffee comes from the species *Coffea arabica* and *Coffea robusta*. Coffee from the arabica species has good flavor and constitutes approximately 80% of the coffee consumed in the world [14]. The composition of coffee changes with the coffee bean species and roast process conditions [41]. Specifically, *Coffea arabica* differs from *Coffea robusta*, and the roast condition differs with time or temperature. Chemical components of green beans in *Coffea arabica* and *Coffea canephora* are shown in Table 2 [16]. Preparations include boiled unfiltered coffee, filtered coffee, and decaffeinated coffee. The compositions differ across the species, degree of roasts and preparation of the coffee. A large number of different compositions are present in coffee, but caffeine, diterpene, kahweol and chlorogenic acid and other phenols are the basic components (Figure 2) [86]. Components with which metabolic syndrome prevention is expected are caffeine, diterpenes, kahweol and polyphenols. In particular, chlorogenic acid has many beneficial effects [69].

**Figure 3:**
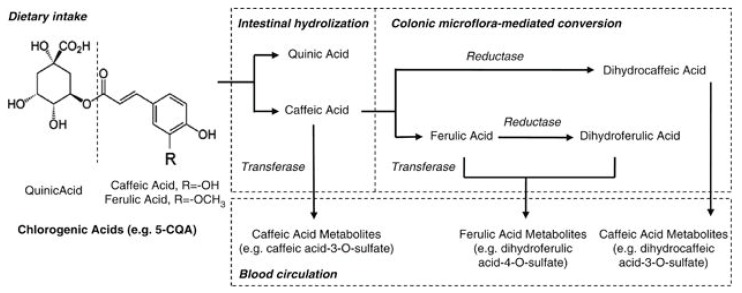
Main metabolic pathway of chlorogenic acids. Dietary chlorogenic acids is hydrolyzed into quinic acid, caffeic and ferulic acid, and further metabolized in small intestine and colon before entering into blood stream (Adapted from reherence Zhao et al., 2012) [87].

Chlorogenic acids are a family of molecules formed between quinic and cinnamic acids and metabolized to several molecules in the body (Figure 3) [87]. The most common chlorogenic acid is 5-*O*-caffeoylquinic acid (CQA), but it is called simply chlorogenic acid. It was reported that the chlorogenic acid content of one 200-mL cup of coffee ranges from 70 to 350 mg [88]. Trigonelline is a niacin-related compound and is another component of coffee. Trigonelline was observed to alter induction of estrogen-dependent growth through the estrogenic action in human breast cancer cells [89]. This activity indicates that trigonelline has an estrogenic effect.

## 5. Chlorogenic Acid and Metabolic Syndrome Associated-Endothelial Dysfunction

The increase of markers of inflammation enhances global cardiovascular risk. The inflammatory response is enhanced early in adipose expansion and chronic obesity during metabolic syndrome onset [27]. Increasing evidence suggests that chronic subclinical inflammation is part of the metabolic syndrome. For example, increased serum concentrations of tumor necrosis factor-α (TNF-α) and IL-6 might attenuate insulin action by inhibiting insulin signaling [88]. A number of features of metabolic syndrome are related to low-grade inflammatory pathological conditions (Table 4) [89]. Increased plasma C-reactive protein has been observed in insulin-resistant and obese subjects and is a surrogate marker for both coronary heart disease and diabetes [90]. The adipose tissue secretes several adipocytokines and induces inflammation and oxidative stress in vascular tissue. In particular, adiponectin and resistin regulate monocyte adherence to vascular endothelial cells [91]. Subsequently, enhancing monocytic migration to the subendothelial space is one of the key events in the development of atherosclerosis. Specifically, the metabolic syndrome induces vascular endothelial cell disorder and induces arteriosclerosis, and arteriosclerosis, in turn increasing the risk for conditions such as myocardial infarction or cerebral infarction. Chlorogenic acid appears to protect normal endothelial activity [92]. On the other hand, chlorogenic acid inhibited interleukin 1 beta (IL-1β)-induced gene expression of vascular cell adhesion molecule-1, intercellular cell adhesion molecule-1 and endothelial cell selectin in human umbilical vein endothelial cells [59]. Also, chlorogenic acid blocked IL-1β-induced nuclear translocation of nuclear factor-kappaB subunits p50 and p65 and suppressed the adhesion of human lymphoma cell line, U937 cells. In addition, a recent study demonstrated the protective effects of chlorogenic acid on human umbilical vein endothelial cells [93]. Namely, chlorogenic acid induced a cell growth higher than those stimulated with inflammatory TNF-α only. Furthermore, chlorogenic acid reduced reactive oxygen species and xanthine oxidase-1 levels, and enhanced superoxide dismutase and heme oxygenase-1 levels in endothelial cells. A study described the effects of chlorogenic acid on endothelial function with oxidant-enhanced damage in isolated aortic rings from mice. Chlorogenic acid reduced HOCl-induced oxidative damage in endothelial cells. The mechanism of the beneficial effect of chlorogenic acid was associated with the production of NO and induction of heme oxygenase-1 [94]. Consumption of coffee with a high content of chlorogenic acids repaired endothelial dysfunction by decreasing oxidative stress [95]. A previous study indicated that oxidative stress has been demonstrated to play a important role in the development of endothelial dysfunction [96]. Chlorogenic acid appears to protect against endothelial dysfunction due to its antioxidant activity. Also, chlorogenic acid inhibited TNFα-induced intercellular adhesion molecule-1, vascular cell adhesion molecule-1, and monocyte chemotactic protein-1 expression in human endothelial cells. In addition, it has been reported that chlorogenic acid blocks α-glucosidase activities, and may thus prevent T2DM [97]. It is suggested that chlorogenic acid prevents vascular endothelial disorder through this inhibitory activity. These results suggest that chlorogenic acid prevents induced atherosclerosis that would otherwise stimulate inflammation. Lysophosphatidylcholine (LPC) is a major phospholipid component of oxidized LDL and is associated with atherogenic induction [98]. A recent report indicated the effects of chlorogenic acid on intracellular calcium control in LPC-treated endothelial cells [99]. Namely, the gene expression of the transient receptor potential canonical (TRPC) channel 1 was enhanced significantly by LPC treatment and inhibited by chlorogenic acid. Thus, chlorogenic acid may protect endothelial cells against LPC injury and inhibit atherosclerosis. 

Furthermore, the effects of the representative chlorogenic acid CQA on vascular function and blood pressure were evaluated in normotensive Wistar–Kyoto rats and spontaneously hypertensive rats [32]. CQA increased NO production and decreased ROS production. In addition, CQA reduced hypertension, and prevented the impairment of endothelial function in spontaneously hypertensive rats. NADPH oxidase-derived superoxide had a important role in the control of vascular tone in health and disease [100]. In endothelial cells, heme oxygenase-1 is induced in response to oxidative stress, which may play a role in vascular prevention. Furthermore, a report demonstrated that the pretreatment of cultured human aortic endothelial cells with 10 μM chlorogenic acid prevented endothelial cell viability following exposure to hypochlorous acid [94]. Chlorogenic acid enhanced endothelial nitric oxide synthase dimerization and induced heme oxygenase-1 protein expression in human aortic endothelial cells. These results are consistent with the endothelial protective effects of coffee consumption. These reports suggest that the coffee component, chlorogenic acid, can protect cultured endothelial cells against inflammation-enhanced endothelial dysfunction and play an important role in the prevention of atherosclerotic complications.

## 6. Conclusions

Metabolic syndrome is a strong risk factor for atherosclerosis-associated CVD and T2DM. Obesity due to excess energy intake strongly enhances the metabolic syndrome—concomitant obesity is the major driver of the syndrome. However, coffee polyphenols can reverse the metabolic risk factors of metabolic syndrome. Coffee polyphenols inhibit atherosclerosis-related CVD and T2DM, respectively. Coffee has many health-promoting properties, and chlorogenic acid appears to protect against metabolic syndrome through its antioxidant activity. The antioxidative effects of coffee components may be a basic feature of prevention.

## Figures and Tables

**Table 1:** Total polyphenol intake from beverages in the Japanese diet ^a^.

Main Food Sources	Consumption of Beverages		Average Total Polyphenol Content	Daily Total Polyphenol Consumption	
Beverages	(mL/day)	(%)	(mg/100 mL)	(mL/day)	(%)
Green tea	353 ± 337	23	115	292 ± 398	34
Coffee	213 ± 213	19	200	426 ± 424	50
Barley tea	174 ± 325	16	9	15 ± 28	2
Oolong tea	76 ± 214	7	39	30 ± 84	4
Fresh milk	60 ± 127	5			
Black tea	59 ± 146	5	96	57 ± 140	7
Other tea	53 ± 182	5	8	4 ± 15	1
Sports drinks	52 ± 180	3			
Carbonated drinks	37 ± 127	3			
Mineral water	35 ± 136	3			
Fruit juice	32 ± 71	3	34	11 ± 24	1
Tomato/vegetable juice	14 ± 56	1	69	9 ± 38	1
Cocoa/chocolate malt drinks	10 ± 49	1	62	6 ± 30	1
Soy milk	6 ± 30	0	36	2 ± 11	0
Others	40 ± 147	4			
Total	1113 ± 512	100		853 ± 512	100

^a^ Adapted from reference Fukushima et al., 2009 [27].

**Table 2:** Comparison of chemical components of green beans in *Coffea arabica* and *Coffea canephora*.

Component	*C. arabica*	*C. canephora*
Minerals *	3.5–4.5	3.9–4.5
Lipids *	13–17	7.2–11
Caffeine *	0.7–2.2 (average 1.4)	1.5–2.8 (average 2.2)
Chlorogenic acid *	4.80–6.14	5.34–6.41
Trigonelline *	1–1.2	0.6–1.7
Oligosaccharides *	6–8	5–7
Total polysaccharides *	50–55	37–47

* % dry matter. Adapted from reference Stamach et al., 2006 [29].

**Table 3:** Characteristics of studies investigating the relationship between coffee consumption and metabolic syndrome and its components.

Design	Population Characteristics	Cases	Diagnosis Criteria	Adjustments	Results	Country	Reference
Cross-sectional	1889 (760 M, 1129 F, mean age 50.2 ± 16.3)	226 (91 M, 135 F)	IDF-MetS	Gender, age, BMI, educational level, socio-economic status, energy intake, smoking status, alcohol drinking, physical activity level, MedDietScore, caffeine, source of caffeine.	Coffee, but not caffeine, was inversely associated with MetS and triglycerides.	Italy	[54]
	8821 (4291 M, 4530 F, mean age 56.8 ± 7)	2461 (1126 M, 1335 F)	IDF-MetS	Gender, age, educational level, occupational level, physical activity, smoking status, alcohol drinking, total energy intake, tea consumption.	Coffee was negatively associated with MetS, WC, hypertension and triglycerides.	Poland	[55]
	17,953 (6879 M, 11,074 F, mean age 39.7, range 19–65)	na	NCEP ATPIII	Age, gender, smoking status, physical activity, alcohol, total energy, education, income.	Comparing ≥3 times/day consumers with those who consumed coffee <1 time/week, the OR for MetS was 1.37, 95% CI 1.10–1.72. In addition, coffee drinkers had an elevated risk of obesity, abdominal obesity and low HDL.	Republic of Korea	[56]
	19,839 (all male, age range 30–79)	3957 (all male)	NCEP APTIII	Age, education level, physical activity, occupation, smoking habits, alcohol habits, dietary factors, and family history of diabetes, hypertension, and cerebrovascular and CVD in second-degree relatives.	Regular drinking of coffee was not associated with MetS.	China	[57]
	554 (409 M, 145 F, mean age 52.2 ± 9.3)	114 (NCEP ATPIII), 77 (JASSO)	NCEP ATPIII/JASSO	Age, gender, total energy intake, physical activity, and smoking and drinking habits.	NCEP ATPIII criteria: Coffee was associated with a lower prevalence of MetS and drinkers of ≥3 cups/day had a lower OR for triglycerides. JASSO criteria: MetS prevalence was not associated with coffee consumption. However 1.5 to 3 cups/day drinkers registered a lower OR for high FPG.	Japan	[58]
	361 (all male, mean age 74.7 ± 6.1)	132 (all male)	Modified NCEP ATPIII	Age, BMI, UA, HOMA-IR, hsCRP, physical activity, psycho-social factors (occupational status, marital status, educational status), alcohol habits, coffee drinking habits.	Coffee drinking was not associated with MetS (OR 0.92, 95% CI 0.27–3.14).	Taiwan	[59]
	3283 (2335 M and 948 F, mean age 46.4, range 20–65)	406 (374 M and 32 F)	JASSO	Age, alcohol drinking, smoking, physical activity.	Coffee consumption of 4 cups or more was protective for MetS (OR 0.61, 95% CI 0.39–0.95), high blood pressure and high triglycerides, when compared with non-coffee drinkers in men. In women, coffee consumption was not associated with the prevalence of MetS or its components.	Japan	[60]
Cross-sectional/prospective	83,436	26,046	Not standard criteria	Age, gender, smoking status, physical inactivity and use of antihypertensive and lipid-lowering medication.	A high coffee intake was associated with low risk of MetS (OR 0.89, 95% CI 0.83–0.95), obesity, type 2 diabetes, high BMI, WC, total cholesterol and low HDL.	Denmark	[51]
Prospective	9514 (1497 M and 5317 F, mean age 53.6 ± 5.7)	3782	AHA	Age, gender, race, education, center, total calories, smoking status, pack-years, physical activity, and intakes of meat, dairy, fruits and vegetables, whole grains, and refined grains.	No relationship was observed between coffee and MetS.	USA	[61]
	17,014 (age range 20–56)	1942	modified NCEP ATPIII	Age, baseline examination, alcohol intake, coffee consumption, number of cigarettes smoked, years of education, leisure-time physical activity.	Coffee intake was not associated with MetS, both in men and women.	Norway	[62]
	368 (174 M and 194 F, mean age 36)	37	NCEP ATPIII	Gender, physical activity, energy intake, smoking behavior, alcohol consumption.	Coffee consumption was not associated with MetS or its components.	Netherlands	[45]
	1902 (785 M and 1117 F, mean age 62.7 ± 11)	188 (137 M and 51 F)	JASSO	Age, gender, total energy intake, alcohol intake, current smoking, and habitual physical activity.	In those with lower coffee consumption there was a higher MetS prevalence, with an inverse relationship between the number of components and coffee consumption. All components of MetS except for HDL-cholesterol were directly associated with coffee.	Japan	[63]
Case–control	250 (103 M and 147 F, age range 18–81)	74 (27 M, 47 F)	NCEP ATPIII	Age, gender, education level, socio-economic status, marital status, hyperglycaemia, chocolate, coffee, milk, sleep.	Coffee was inversely associated with metabolic syndrome.	Brazil	[47]

*Abbreviations*: AHA: American Heart Association; BMI: body mass index; CI: confidence interval; CVD: cardiovascular disease; HDL: high-density lipoprotein; HMW-Ad: high-molecular-weight serum adiponectin; HOMA-IR: homeostasis model-insulin resistance index; hsCRP: high-sensitivity C-reactive protein; IDF: International Diabetes Federation; JASSO: Japan Society for the Study of Obesity; MetS: metabolic syndrome; na: not available; NCEP ATPIII: National Cholesterol Education Program Adult Treatment Panel III; OR: odds ratio; WC: waist circumference. Adapted from reference Marventano et al., 2016 [12].

**Table 4:** The inflammatory component of the metabolic syndrome ^a^.

Vascular dysfunction	Endothelial dysfunction
	Microalbuminuria
Proinflammatory state	Elevated high sensitivity C-reactive protein and serum amyloid A
	Elevated inflammatory cytokines (TNF-α, IL-6)
	Decreased adiponectin levels
Prothrombotic state	
Insulin resistance	
Visceral adiposity	

Abbreviations: IL-6, interleukin 6; TNF-α, tumor necrosis factor-α. ^a^ Adapted from reherence Paoletti et al., 2006 [90].

## Abbreviations

BMI	body mass index
CVD	cardiovascular disease
CQA	5-*O*-caffeoylquinic acid
EPR	electron paramagnetic resonance
IL-1β	interleukin 1 beta
LDL	low-density lipoprotein
LPC	lysophosphatidylcholine
NADPH	nicotinamide adenine dinucleotide phosphate
NAFLD	non-alcoholic fatty liver disease
NO	nitric oxide
ROS	reactive oxygen species
T2DM	type 2 diabetes
TNF-α	tumor necrosis factor-α
